# Genetic Association Studies of Copy-Number Variation: Should Assignment of Copy Number States Precede Testing?

**DOI:** 10.1371/journal.pone.0034262

**Published:** 2012-04-06

**Authors:** Patrick Breheny, Prabhakar Chalise, Anthony Batzler, Liewei Wang, Brooke L. Fridley

**Affiliations:** 1 Department of Biostatistics, University of Kentucky, Lexington, Kentucky, United States of America; 2 Department of Biostatistics, University of Kansas Medical Center, Kansas City, Kansas, United States of America; 3 Department of Health Sciences Research, Mayo Clinic, Rochester, Minnesota, United States of America; 4 Department of Molecular Pharmacology and Experimental Therapeutics, Mayo Clinic, Rochester, Minnesota, United States of America; Emory University School Of Medicine, United States of America

## Abstract

Recently, structural variation in the genome has been implicated in many complex diseases. Using genomewide single nucleotide polymorphism (SNP) arrays, researchers are able to investigate the impact not only of SNP variation, but also of copy-number variants (CNVs) on the phenotype. The most common analytic approach involves estimating, at the level of the individual genome, the underlying number of copies present at each location. Once this is completed, tests are performed to determine the association between copy number state and phenotype. An alternative approach is to carry out association testing first, between phenotype and raw intensities from the SNP array at the level of the individual marker, and then aggregate neighboring test results to identify CNVs associated with the phenotype. Here, we explore the strengths and weaknesses of these two approaches using both simulations and real data from a pharmacogenomic study of the chemotherapeutic agent gemcitabine. Our results indicate that pooled marker-level testing is capable of offering a dramatic increase in power (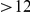-fold) over CNV-level testing, particularly for small CNVs. However, CNV-level testing is superior when CNVs are large and rare; understanding these tradeoffs is an important consideration in conducting association studies of structural variation.

## Introduction

The analysis of individual human genomes has revealed an unexpected amount of variability in the form of duplication and deletion of genomic regions [1], [2]. Since then, a number of studies have shown that copy-number variation plays a large role in genetic diversity [3], [4]. Other studies have identified associations between copy-number variation and various diseases, including Crohn's disease, psoriasis, schizophrenia, and autism [5]–[9].

Because humans have two copies of each chromosome, most individuals have two copies of a given genomic segment. Copy-number variation exists when an individual has one or more deletions or duplications of that segment, resulting in a different number of copies. Such an individual is said to possess a CNV at that region, while the normal, or copy-neutral, individuals do not.

This article discusses statistical approaches for conducting genetic association studies of copy-number variation. Such studies attempt to identify associations between a phenotype, such as disease state or drug response phenotype, and genetic variation in the form of changes in copy number. There are several techniques for measuring copy-number variation, including array comparative genomic hybridization and next-generation sequencing. We focus here on the detection of copy-number changes using data from genomewide single nucleotide polymorphism (SNP) arrays, although many of the issues that we explore are relevant regardless of the platform used to obtain the copy-number measurements. The main advantage of SNP arrays is that vast amounts of this type of data have already been collected in pursuit of identifying associations involving SNPs. The convenience and low cost of re-analyzing these data for copy-number variation has prompted a number of studies, and should continue to do so for years to come.

We compare two strategies for CNV association testing. Each strategy consists of two stages. In the first approach, which we refer to as *CNV-level testing*, stage I consists of estimating the number of copies present at all segments of the genome, for each individual. This is often referred to as “CNV calling.” Next, stage II consists of carrying out a genetic association test at every segment for which copy-number variability exists. A number of articles [10]–[13] have compared various methods for CNV calling. Our approach here is different; we are interested in comparing this family of approaches with an entirely different approach which does not involve CNV calling.

In this second approach, which we refer to as *marker-level testing*, stage I consists of carrying out an association test at every genetic marker using raw intensity data from the SNP array. Since CNVs span multiple markers, the presence of a single CNV that affects the phenotype will elevate the test statistics for several nearby markers. This is the motivation for stage II, which consists of pooling test results across neighboring markers to determine CNV regions associated with the phenotype.

Because the above approaches consist of two stages, each approach risks losing information in the first stage that may diminish power in the second stage. We illustrate that this is indeed a concern, and furthermore, that the type of information lost by each approach is quite different. This has strong implications for the power of each method to detect various forms of CNV-phenotype associations. After a more detailed description of the data from such studies, we illustrate the two approaches and then compare them using both real and simulated data.

## Methods

### Data

The data in this article comes from a pharmacogenomic study of gemcitabine, a commonly used treatment for pancreatic cancer. In this section, we describe the design of the study, the general characteristics of data arising from such studies, and how this data was used to create spike-in simulated data sets which allow us to estimate and contrast the power of the CNV- and marker-level testing approaches.

### Gemcitabine pharmacogenomic study

The gemcitabine pharmacogenomic study [14], [15] was carried out on the Human Variation Panel (HVP), a cell based model system. The HVP consists of EBV-transformed B lymphoblastoid cells derived from Caucasian-American (CA), African-American (AA) and Han Chinese-American (HCA) subjects (Coriell Institute, Camden, NJ). Gemcitabine drug cytotoxicity data were collected at eight drug dosages (1000, 100, 10, 1, 0.1, 0.01, 0.001, and 0.0001 uM) [14]. Estimation of the drug response phenotype IC50 (the effective dose that kills 50% of the cells) is then completed using a four parameter logistic model [16]. Genotyping of markers for the cell lines was completed using the Illumina HumanHap 550K and HumanHap510S at the Genotyping Shared Resources at the Mayo Clinic in Rochester, MN, which consists of a total of 1,055,048 markers [15], [17]. In addition to the called genotypes for the SNP markers, we have the raw intensity data to be used in CNV analysis. One hundred seventy-four cell lines (60 Caucasian, 54 African American, 60 Han Chinese American) had both gemcitabine cytotoxicity measurements and genome-wide marker intensity data. To compare the two approaches for CNV analysis for a pharmacogenomic study, we chose one chromosome (chromosome 3) from the genome-wide data. Raw data was normalized according to the procedure outlined in Barnes *et al.* (2008) [18], which corrects for a number of potential biases, including batch effects and differences in hybridization intensity among the probes. To control for the possibility of population stratification, which can lead to spurious associations, we used the method developed by Price *et al.* (2006) [19], which uses a principal components analysis to adjust for stratification.

General structure of CNV data from SNP arraysThe raw data that arises from the gemcitabine study, or any similar study involving genome-wide SNP arrays such as those manufactured by Illumina or Affymetrix, consists of two intensity measurements for each SNP, corresponding to the A and B alleles [20], [21]. These intensities are then transformed into polar coordinates, with 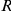 representing the overall intensity and 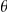 representing the relative contribution from each allele. To account for systematic differences in intensity between the two alleles, one considers the ratio between 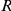 and the expected value of 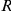 given neutral copy number. Finally, a log transformation is applied. The result (the log 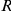 ratio, or LRR) serves as a continuous measurement of copy number and is vaguely normal in distribution, though with thicker tails. In addition to SNP markers, many genotyping arrays now include non-polymorphic markers specifically for the purpose of copy-number measurement. We use the generic term *marker* to refer to any position on the genome in which an intensity measurement is obtained.

**Figure 1:**
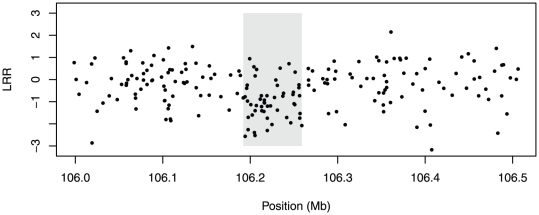
Example of LRR data for a putative CNV on Chromosome 3 for a cell line in the gemcitabine study. The gray region denotes the estimated boundary of the CNV. The points in the gray region have a mean LRR of −0.98; the surrounding points have a mean of −0.11.

An illustration of the what this type of data looks like in the presence of a putative CNV is presented in Figure 1. As the figure illustrates, there is a substantial amount of noise present in the data relative to the magnitude of the shift in LRR. Because of this noise, the drop in LRR may not be obvious at a glance. However, the statistical evidence is fairly convincing: a 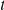-test of whether the mean LRR for the markers in the gray region is equal to that of the surrounding markers has 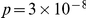. Clearly, there is a need for good statistical methods to distinguish signals from noise.

### Spike-in simulations

In order to study the power of CNV- and marker-level testing approaches, we must be able to simulate CNVs and their corresponding LRR intensity measurements. The accuracy of these power estimates is affected by how realistic the simulated data is, so we give careful thought here to simulating this data in as realistic a manner as possible. The basic design of our simulations is use real data from the gemcitabine study and “spike” in a signal, then observe the frequency with which we can recover that signal. We used circular binary segmentation (described in “CNV-level testing”) to estimate each sample's underlying mean LRR at every position along the chromosome, then subtracted the estimated mean from the actual LRR measurement to obtain a matrix of residuals representing the noise accompanying the measurement of LRR. We restricted this effort to chromosome 3 of the gemcitabine pharmacogenomic study, resulting in a residual matrix containing 172 samples and 70,542 markers.

We then used these residuals to simulate LRR noise over short genomic regions in which a single simulated duplication is present. Letting 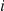 denote subjects and 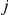 denote markers, the following variables are generated: 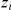, an indicator for the presence or absence of a CNV in individual 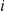; 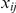, the LRR measurement at marker 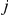 for individual 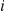; and 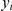, the phenotype. For each simulated data set, 200 markers were randomly selected from chromosome 3. The LRR measurement error for simulated subject 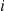 was then taken from the observed measurement errors at those markers for a randomly chosen cell line in the data from the gemcitabine study. The random selection of markers from throughout the chromosome removes the possibility of bias arising from correlation among the intensities of nearby markers, which otherwise may arise from missed CNVs during the CBS estimation or genomic “waves” caused by local variation in genomic GC content [22], [23].

Thus, within a simulated data set, all subjects are studied with respect to the same genetic markers, but the markers vary from data set to data set. Simulating the data in this way preserves all the features of outliers, heavy-tailed distributions, skewness, unequal variability among markers, and unequal variability among subjects that are present in real data. A 200 marker region corresponds to, on average, a 560 kb region of chromosome 3. We varied the length of the CNV from 10 to 50 markers, corresponding to a size range of 26 to 137 kb. For the Illumina Human1M-Duo BeadChip, which has a median spacing of 1.5 kb between markers, these numbers of markers would correspond to simulating a 300 kb region with CNV size ranging from 15 to 75 kb.

We simulate results from two study designs: a population-based cohort study in which the outcome is continuous and a case-control study in which the outcome is binary. In the cohort study, the CNV indicator, 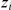, is generated from a Bernoulli distribution, where 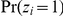 is the frequency of the CNV in the population; subsequently, 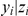 is generated from a normal distribution whose mean depends on 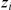. In the case-control study, the outcomes are fixed (in our simulation, half of the subjects were cases and the other half controls), whereas 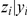 is generated from a Bernoulli distribution with a probability given by Bayes' rule that depends on the frequency of the CNV in the population (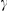), the prevalence of the disease in the normal population (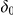), and the penetrance of the genetic mutation (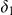):
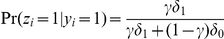


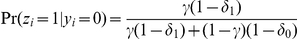
Note that in both sampling designs, the phenotype and LRR are conditionally independent given the latent copy-number status 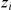.

**Figure 2:**
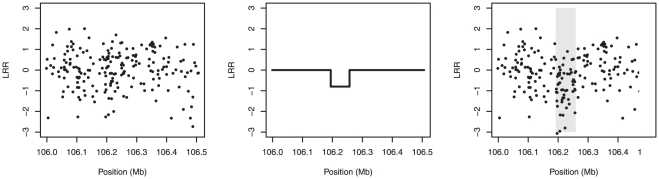
Example of LRR data for a simulated CNV. *Left:* The noise, randomly drawn from among the observed measurement errors for a single subject. *Middle:* The spiked-in signal. *Right:* The resulting simulated data, which looks qualitatively similar to the real CNV in Figure 1.

As mentioned earlier, the LRR values, 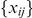, derive from the observed residuals in the real data. To this noise, we add a signal that depends on the presence of the simulated CNV, 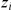. The added signal is equal to zero unless the simulated genome contains a CNV encompassing the 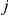
th marker; otherwise the added signal is equal to the standard deviation of the measurement error times the signal to noise ratio. Our simulations employed a signal-to-noise ratio of 0.8, which corresponded roughly to a medium-sized detectable signal based on our inspection of the gemcitabine data. An illustration of the spike-in process is given in Figure 2.

For the simulations presented in the remainder of the article, we used a sample size of 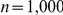. For continuous outcomes, we used an effect size (change in mean divided by standard deviation) of 0.4. For the case control studies, we assumed a rare disease (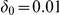) and a CNV which confers a relative risk of 2. All association tests were conducted with a nominal overall type I error rate of 0.05.

### CNV-level testing

The idea behind CNV-level testing is to first separate each individual's genome into regions of constant copy number and then to use those results for subsequent association testing. Thus, the first challenge is to develop a method for detecting departures from copy-neutral status.

Several methods have been proposed for this task; among the most prominent are hidden Markov models [24], [25], circular binary segmentation [26], [27], and the fused lasso [28], [29]. We focus here on circular binary segmentation, which has been found to compare favorably with other methods [10], [11]. However, as we will comment on in the discussion, our main conclusions regarding the fundamental differences between CNV-level and marker-level tests would likely apply to the other methods as well.

The main idea behind circular binary segmentation (CBS) is as follows. For each chromosome,

1. Form the sequence of LRR intensities into a circle by joining the first and last markers
2. For all possible ways of dividing up the circle into complimentary arcs, compute the t-test statistic for a difference in means between the two arcs
3. If the maximum of these test statistics exceeds its null distribution critical value, segment the circle there
4. Repeat recursively for the segmented arcs until no more significant segments can be found

To carry out this analysis, we used the R package DNAcopy (available at http://www.bioconductor.org/packages/release/bioc/html/DNAcopy.html), which obtains the critical values in step 3 above using a permutation testing approach. For details of this procedure and its implementation, see [26], [27]. The output of this procedure is an estimation, at every position along the genome, of the mean LRR at that position. These estimates, which we denote 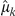, are piecewise constant over arc 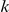, and therefore also provide an estimation of the CNV structure of each individual's genome.

Once these estimates have been obtained, the second stage in CNV-level testing is to carry out the association test. In practice, this can be fairly complicated, for at least three reasons: (1) the test can be based on either a continuous measure, 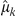 or a discretization such as whether 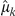 represents a duplication (gain), deletion (loss), or normal value. (2) Overlapping CNVs do not necessarily share the same boundaries. Whether or not these partially overlapping CNVs represent the same CNV or different CNVs can be a rather complicated decision, especially when the sample size is reasonably large, as the number of overlap patterns can be considerable. (3) Because CNVs from different individuals do not overlap perfectly, the CNV-level tests are correlated; this complicates efforts to correct for multiple testing. For our simulations, we avoid these complications by focusing only on a small genomic region with a single CNV and basing the test on whether a CNV is detected or not, thereby skirting the above complications. We then conduct either a 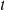-test or Fisher's exact test, depending on whether the phenotype is continuous or binary. However, it is worth noting that applications of CNV-level tests to actual, genomewide data must contend with the above three issues; this is discussed further in the Results section.

An important consideration in the use of CBS for subsequent association testing is the threshold used to declare a CNV present. If the threshold is too high, true CNVs will go undetected; if this threshold is too low, false positives will occur as neutral regions are called as CNVs. The tradeoff between false positives and false negatives depends on the frequency of the CNV, as Table 1 demonstrates.

**Table 1:** Effect of CNV-calling threshold (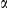) on the power to detect a CNV.

		Calling		
		threshold		
		0.001	0.01	0.1
**CNV**	**5%**	**17.9**	**29.0**	**22.1**
**Frequency**	**10%**	**32.5**	**55.4**	**52.8**
	20%	50.0	82.0	88.8

Continuous outcome, 10,000 replications per cell, CNV size = 30 markers).

As the table shows, false positives are a larger problem when the CNV is rare than when it is common. While highly stringent false positive rates of 0.001 and 0.01 perform well when the CNV is rare (5% frequency in the population), the more liberal critical value of 0.1 attains the best power when the CNV is common (20% population frequency). This is not surprising. One would anticipate that power is roughly proportional to misclassification rate; misclassification rate in turn is dominated by false positives when CNVs are rare. For more common CNVs, however, highly stringent thresholds cause problems as false negatives become frequent.

In any real study, of course, there will presumably be a mixture of common and rare CNVs that may be associated with the phenotype. The above results indicate that a threshold of 0.01 is fairly robust over a realistic range of CNV frequencies. This trend was observed across a range of different marker sizes (data not shown); accordingly, we use this value for subsequent simulations involving CNV-level tests.

### Marker-level testing

**Figure 3:**
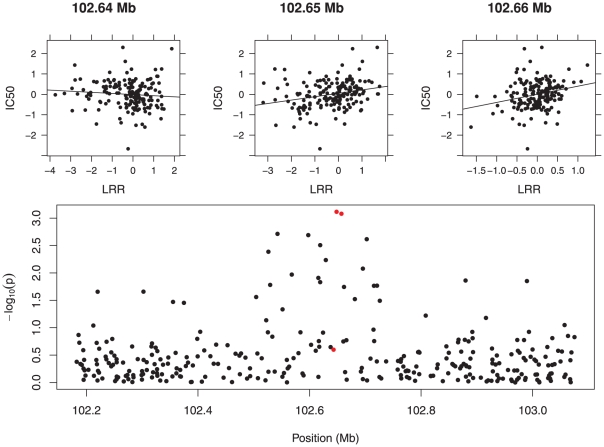
Illustration of marker-level testing. *Top:* Marker-level tests at three markers for the gemcitabine study. The phenotype (IC50, a continuous outcome described in “Gemcitabine pharmacogenomic study”) is plotted as a function of LRR, along with the regression line. The 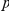-values for the three 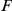-tests are, respectively, from left to right. 0.25, 0.0008, and 0.0008, respectively, from left to right. *Bottom:* Plot of 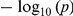 from the marker-level tests as a function of position along the chromosome. The three tests from the top part of the figure are plotted in red.

A lesser known alternative to CNV-level testing is marker-level testing, in which association testing between copy number and phenotype is carried out at the level of the single marker. These tests make no effort to call CNVs as present or absent; instead, they utilize intensity as a continuous measurement of copy number at each marker. For example, if our phenotype is continuous, each marker-level test may derive from a linear regression model. Such a model may involve adjustments for additional factors, such as race and age. Figure 3 illustrates the basic idea: three marker-level tests are depicted, as well as a plot of the resulting 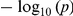 values along the chromosome. As the figure illustrates, each individual test is not particularly convincing due to the high variability of the LRR measurements, but the aggregation of a large number of tests with low 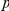-values in close genetic proximity to each other strongly suggests a copy number-phenotype association.

The second stage in marker-level testing is to identify these regions in which low 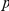-values have aggregated. This requires a systematic method for pooling information across neighboring hypothesis tests. We propose here to pool test results by using circular binary segmentation (described in “CNV-level testing”) on the 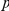-values. Certainly, there are other possibilities (see Discussion). Our purpose in this paper, however, is to broadly illustrate the strengths and weaknesses of marker-level testing versus CNV-level testing, and to that end we restrict attention here to CBS as the method by which the genome is segmented in both testing approaches.

One factor we do explore, however, is the effect of transforming the 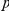-values. In particular, one may imagine performing CBS on the 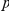-values themselves, on 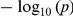, or on 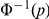, where 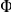 is the CDF of the standard normal distribution. The last transformation (the so-called “probit” transformation) is motivated by the idea that the resulting quantity will follow a normal distribution under the null, which should provide maximum power in the subsequent 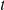-tests performed by CBS. The increase in power that results from using this transformation is illustrated in Table 2, based on simulation results. We therefore use the probit transformation for marker-level testing when comparing association testing approaches in the Results section. It is worth noting that the 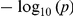 transformation is highly sensitive to low 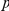-values. Although this is seemingly attractive, it decreased the power. This raises the question of whether some other method of marker-level test aggregation might be able to harness this feature more effectively than CBS. This is an interesting question for future research, but beyond the scope of this paper to address.

**Table 2:** Effect of various transformations of 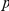-value prior to application of CBS on the power to detect a CNV.

		No	Transformation		
		pooling	None	Probit	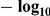
**CNV**	5%	6.8	8.6	8.6	6.7
**frequency**	10%	15.2	30.8	34.2	24.1
	20%	54.0	82.9	88.0	76.8

Continuous outcome, 10,000 replications per cell, CNV size = 30 markers. “Power” here refers to the probability that a segment in which low 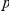-values have aggregated can be separated from the test results from surrounding markers. The “no pooling” analysis (which implements a Bonferroni correction to maintain the correct overall type I error rate) is included to illustrate the power gained by pooling information across nearby markers.

## Results

### Simulated data

Using the simulation setup described in “Spike-in simulations”, we compared the power of both CNV- and marker-level approaches while varying study design, CNV prevalence, and CNV size. For each setting, 10,000 independent data sets were generated and analyzed. Power was defined as the fraction of time a CNV-phenotype association was declared. Note that this does not take into account fraction of overlap. Certainly, one would prefer a method that not only detects CNV associations but correctly identifies their boundaries; we focus only on yes/no detection of copy-number association here.

**Figure 4:**
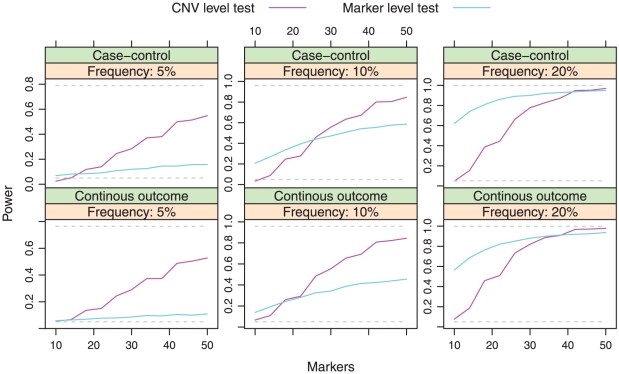
Power as a function of method and CNV size. The CNV-level testing approach uses a false positive CNV call rate of 0.01; the marker-level approach uses the probit transformation. The lower dashed line represents the type I error rate, while the upper dashed line represents the “oracle” power that would be possible if the true CNV status were known, with no measurement error.

In the absence of spiked-in signal, each approach preserved the type I error rate of 5% for both study designs. The power of each approach to detect genetic associations in the presence of a spiked-in, causative CNV is illustrated in Figure 4. The figure illustrates a very interesting contrast between the two approaches. Relative to marker-level testing, CNV-level testing works better for large, rare CNVs. On the other hand, marker-level testing performs better when CNVs are smaller and more common. Both methods detect associations involving large, common CNVs with adequate power, while neither method was able to detect small, rare CNVs – note that for both approaches, the power drops to the nominal type I error rate of 5% as the limit of reliable detection is approached.

This is an intuitive finding (at least in retrospect). CNV calling pools information across markers. This is most effective for large CNVs. Typically, however, methods for CNV calling do not make use of CNV frequency; this is valuable information when CNVs are common. Marker-level testing does the opposite, pooling information across subjects before attempting to identify significant CNVs. This is most effective when the CNV is common. However, the marker-level approach does not take advantage of the information provided by neighboring markers when conducting its initial tests, which is valuable information for detecting large CNVs.

How large? How rare? In our simulations, marker-level testing had low power to detect associations involving CNVs occurring in 5% or fewer of the subjects, while CNV-level testing had little power to detect associations involving CNVs consisting of fewer than 20 markers (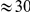 kb). However, these results provide no more than a rough guide. Different ways of carrying out these two approaches (such as using hidden Markov models instead of CBS) or using different types of SNP arrays will likely affect the precise number of markers at which one approach becomes better than the other. The important point is that over the range of biologically plausible values, neither CNV testing approach is clearly superior. Indeed, the differences in power can be considerable. For 10-marker CNVs that occur with 20% frequency, marker-level testing was found to be over 12 times more powerful (62% vs. 5%; case-control results), whereas for 50-marker CNVs with 5% frequency, CNV-level testing was found to be 4.8 times more powerful (53% vs. 11%; continuous outcome results).

### Gemcitabine study

The data were analyzed using both the pooled marker-level testing approach and the CNV-level testing approach. To deal with the issue of partial overlap among CNV calls, we used the cghMCR package (available at http://www.bioconductor.org/packages/release/bioc/html/cghMCR.html) to find minimal common regions among the CNV calls [30]. Minimal common regions with at least three shared gains or losses among cell lines in the sample were considered for subsequent association testing. The most widely shared common region consisted of 20 cell lines with a CNV in that region.

To account for multiple comparisons with the CNV-level testing approach, false discovery rates [31], [32] were calculated. This is somewhat conservative, as partially overlapping CNVs across cell lines introduce dependence across the tests, thereby reducing the effective number of independent tests. Accounting for multiple comparisons is more straightforward with marker-level testing, as the approach we outline in “Marker-level testing” directly controls the family-wise error rate (FWER) of the overall procedure (in the weak sense [33]).

The marker-level approach identified 8 distinct regions at a chromosome-wide significance level of 0.05. At a false discovery rate of 5%, the CNV-level approach identified three regions associated with IC50. Neither of these regions overlapped with the marker-level regions (Table 3). There were, however, regions for which the two approaches demonstrated modest agreement (51.4 Mb and 199.3 Mb), albeit not at the level of 5% chromosome-wide significance.

**Table 3:** Comparison of CNV-level and marker-level tests for the Gemcitabine data.

		CNV-level			Marker-level	Other
	Position (Mb)	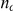	p	q	FWER	studies
Detected by CNV-level approach	12.3–12.45	3	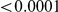	0.03	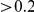	
	51.4–51.5	7	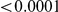	0.01	0.1–0.2	[34]–[36]
	185.1–185.2	3	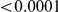	0.01	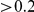	[35], [37]–[39]
	11.3–11.5	3	0.7	0.9	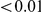	[35], [37], [40], [41]
Detected by marker – level approach	41.78–41.80	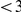			0.01–0.05	[36], [42]
	42.6–42.7	4	0.4	0.8	0.01–0.05	
	44.2–44.4	3	0.1	0.6	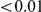	[36]
	102.5–102.7	6	0.4	0.8	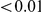	
	132.5–132.6	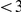			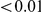	[34], [43]
	139.6–139.8	5	0.2	0.6	0.01–0.05	
	199.28–199.32	4	0.02	0.3	0.01–0.05	[1], [3], [34], [35], [37], [40], [44]–[48]

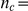
number of CNV calls in that region. If 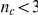, no association test was carried out, hence the blank entries. For the marker-level tests, a FWER of 0.01–0.05 means that controlling the FWER at the 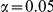 level, we obtain a segmentation in this region, but that if we control the FWER at the 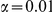 level, we do not.

**Figure 5:**
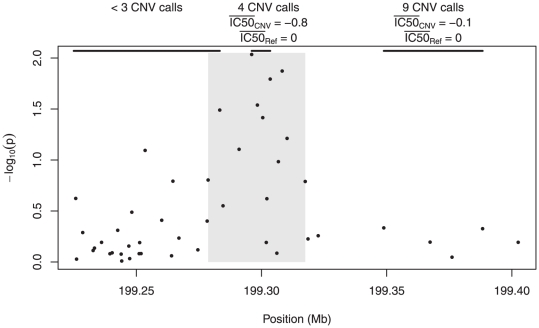
Plot of 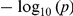 from Gemcitabine marker-level tests as a function of position along the chromosome. The shaded region denotes a region of significant elevation, as detected by the methods described in “Marker-level testing”. The top of the plot contains annotations describing the results of the CNV-level analysis in three distinct regions. 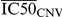 is the mean adjusted IC50 for cell lines with a called CNV in that region; 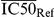 is the mean adjusted IC50 for cell lines without a called CNV in that region.

We take a closer look at the region spanning 199.28–199.32 Mb in Figure 5. This region contained 15 markers, 6 of which had marker-level 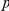-values below .05. At the top of the figure, the CNV-level testing approach is depicted. In the middle of the region, CNV calls were made for four cell lines. These lines had a mean adjusted IC50 of −0.8, quite a bit below the mean of 0 for the lines without a called CNV in that region (the adjustment procedure described in “Gemcitabine pharmacogenomic study” centers the response to have an overall mean of 0). A Wilcoxon rank-sum test comparing the two groups has a 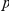-value of 0.02, suggesting an association between the CNV and Gemcitabine cytotoxicity that is in agreement with the one discovered by the marker-level approach. For CNV-level testing, however, evidence for the association is weak after adjusting for multiple comparisons.

To the left of the shaded region in Figure 5, the marker-level approach shows no evidence of association, and indeed, no common regions were found there (and hence, no association test was carried out). Meanwhile, to the right of the shaded region, which also showed no evidence of association in the marker-level approach, a common region consisting of nine cell lines was found. However, this region showed no association with the outcome: the mean adjusted IC50 was nearly the same for those lines with a CNV in the common region and those without (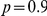).

Not all regions demonstrated this level of agreement. For example, consider the region 102.52–102.73 Mb, plotted at the bottom of Figure 3. It is obvious even to the naked eye that some sort of association is present, and yet no common regions were observed in this span of the chromosome. Clearly, there seems to be information present in the continuous LRR measurements that is lost when an attempt at CNV calling is made. One possibility is that this region harbors a number of small variants that cannot be detected by CNV calling due to an insufficient number of markers per CNV, but that do not stand in the way of detection using the single-marker approach.

There were also regions detected by the CNV-level approach and not the marker-level approach, such as the region 12.31–12.45 Mb. This region has a large number of markers (241), making the individual CNVs easy to recognize and call. Furthermore, the two cell lines with the highest IC50 levels both had CNVs in this region, and the CNV-level association test was highly significant (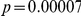, 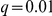). However, the CNV was also rare, present in only 3 out of 172 cell lines. The simulation results demonstrate that the marker-level approach has lower power that the CNV-level approach when the CNV is rare, which helps to explain why no association was found in this region using the marker-level approach.

CNV analysis of the of the gemcitabine pharmacogenomics study involving the Human Variation Panel has also been carried out by Kalari *et al.* [49]. In their analysis, they discovered 775 CNVs with allele frequencies 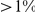 in 102 regions across the genome, including 12 regions on chromosome 3. Using a CNV-level testing approach, they reported five CNV regions to be associated with gemcitabine IC50; none, however, were located on chromosome 3.

## Discussion

We have explored two different approaches to testing for associations between copy number and phenotype. Our results show that CNV-level testing has greater power to detect associations involving large, rare CNVs, while marker-level testing has greater power to detect associations involving small, common CNVs.

Of course, there are other concerns besides power. Plots such as those in Figures 3 and 5 may be of descriptive interest regardless of the formal approach to association testing used. Circular binary segmentation is rather computationally intensive, and is the primary computational burden in the analysis. In a CNV-level analysis, CBS must be run 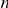 times (once for each subject), whereas in a marker-level analysis, it needs to be run only once (on the 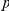-values). For our analysis of the gemcitabine data in the Results section, carried out on an Intel 3.00 GHz processor, the marker-level analysis required 22.6 seconds, while the CNV-level analysis required 52.5 minutes. Furthermore, issues of partially overlapping CNVs and correction for multiple testing are far more complicated and challenging in the CNV-level approach than the marker-level approach.

We used a relatively simple method (CBS) for CNV calling in this study. There are a variety of competing tools, and indeed, this is an active area of methodological development. Certainly, the specific numbers in the power calculations would differ for other CNV calling tools. However, the main message of this article is the general trend and fundamental differences between the CNV-level and marker-level approaches, regardless of the specific techniques used for CNV calling or marker-level test aggregation.

Indeed, as marker-level approaches are less well-known in the statistical genetics community, far less work has gone into developing methods for them, and there is undoubtedly much room for improvement using marker-level approaches beyond the simple approach presented here. Alternative approaches include hidden Markov models, the fused lasso, local regression and kernel-based approaches [50]–[54]. Nor is it clear that pooling 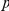-values is the best approach; a more powerful approach may be to pool test statistics instead of 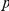-values to account for the direction of the association. Further research is needed to compare the relative merits of these approaches.

Furthermore, our simulations involve a very simple genetic scenario: a small segment of DNA in which a single CNV is either present or absent. It is important to understand the properties of CNV- and marker-level approaches in these simple cases, although future research involving more complicated scenarios is also needed.

Our findings are important for two reasons. First, as both of these approaches are used in practice, it is important for researchers to be aware of their strengths and limitations for detecting certain kinds of CNV-phenotype associations. In practice, the genetic mechanism is unknown, and may be due to rare, large CNVs or small, common CNVs – or a combination of both. An over-reliance on either approach is likely to lead to missing certain types of associations, as we observed in our analysis of the gemcitabine data.

Second, these findings highlight the inadequacy of current approaches and the need to develop methods capable of simultaneously pooling information across both markers and subjects for CNV detection and association studies. Indeed, several recent articles have proposed methods along those lines [55]–[57]. Such methods have the potential to avoid the loss of power and information that comes from current two-stage approaches and deliver robust power to detect the wide variety of CNV-phenotype associations that may exist in nature.
